# Modulation of local immunity by the vaginal microbiome is associated with triggering spontaneous preterm birth

**DOI:** 10.3389/fimmu.2024.1481611

**Published:** 2024-11-18

**Authors:** Yijia Liang, Changying Zhao, Yan Wen, Dashuang Sheng, Tiantian Wei, Tianqi Hu, Junhui Dai, Guoping Zhao, Sijie Yang, Qinghua Wang, Lei Zhang

**Affiliations:** ^1^ Microbiome-X, School of Public Health, Cheeloo College of Medicine, Shandong University, Jinan, China; ^2^ Reproductive Medicine Center, Shandong Provincial Maternal and Child Health Care Hospital Affiliated to Qingdao University, Jinan, China; ^3^ Qingdao West Coast New District Health Bureau, Qingdao, China; ^4^ State Key Laboratory of Microbial Technology, Shandong University, Qingdao, China; ^5^ CAS Key Laboratory of Computational Biology, Bio-Med Big Data Center, Shanghai Institute of Nutrition and Health, University of Chinese Academy of Sciences, Chinese Academy of Sciences, Shanghai, China; ^6^ State Key Laboratory of Reproductive Medicine and Offspring Health, Center for Reproductive Medicine, Institute of Women, Children and Reproductive Health, Shandong University, Jinan, China; ^7^ School of Biological Science and Technology, University of Jinan, Jinan, China

**Keywords:** preterm birth, vaginal microbiome, immunity, *Gardnerella vaginalis*, *Lactobacillus crispatus*

## Abstract

**Objective:**

This study aimed to identify immune states associated with a high risk of preterm birth by immunophenotyping in pregnant populations, and to elucidate the characteristics of immune subtypes and their relationships with preterm birth. Additionally, it sought to uncover the microbial composition and functional characteristics of immune states linked to preterm birth, and to evaluate the impact of bacterial interactions on the initiation of preterm birth.

**Methods:**

Utilizing 16S rRNA sequencing data and local immune factor expression data from a publicly available longitudinal pregnancy cohort, we conducted immunophenotyping through unsupervised clustering of the immune factors. We compared the differences in vaginal microbiota richness, diversity, and composition between identified immune subtypes using α and β diversity analysis. Signature microbiotas were identified using LEfSe analysis, and functional pathway enrichment variations were analyzed using PICRUSt2. Bidirectional mediation analysis was employed to construct a network of mediating roles, and preliminary *in vitro* validation of the Microbial-Cytokine-Preterm Birth pathway was performed to explore the effects of microbial and immune characteristics on vaginal epithelial cell function.

**Results:**

Pregnant women were categorized into three immune subtypes based on local immune status. Microbial functional analysis identified 31 distinct functional pathways, six of which were downregulated in the preterm birth and excessive inflammatory response group. Significant differences in vaginal microbial diversity and composition were observed among pregnant women with different immune subtypes. Bidirectional mediation analysis revealed multiple intermediary roles in preterm birth, highlighting C3b/iC3b and IL-8 in mid-pregnancy and IgE and IgM in late pregnancy.

**Conclusion:**

This study classified pregnant women into three immune subtypes, with the excessive inflammatory response subtype showing a higher predisposition to preterm birth. Mid-pregnancy immune status emerged as a key indicator of preterm birth risk, associated with the vaginal microbiome composition. Microorganisms affected the occurrence of preterm birth by modulating immune factor levels, with time-specific mediation roles observed. *Lactobacillus crispatus* demonstrated potential in protecting against preterm birth by modulating vaginal immune status.

## 1 Introduction

Preterm birth (PTB), defined as birth before 37 completed weeks of gestation, is the leading cause of death among neonates and children under the age of 5, posing a significant threat to the health and quality of life for both mothers and infants (1). The global preterm birth rate was estimated at over 10% (2). Despite several decades of research, there have been few significant breakthroughs in the prediction, prevention, and treatment of spontaneous preterm birth (sPTB) (3). This is mainly because sPTB is a multifactorial syndrome with potential underlying causes ranging from single to multiple factors. The inability to predict and understand the causes of sPTB limited effective interventions and treatments.

There has been a surge in studies implicating a role of vaginal microbiota in sPTB (4–8), but most are associative without mechanistic insight (3, 7–10). For example, the environment dominated by *Lactobacillus crispatus* during pregnancy is often reported as a driver of a healthy pregnancy (6, 11), while the more diverse environments are associated with sPTB (12). Many studies have also shown significant differences in the vaginal microbiome profiles of women of different ancestries. The distinct taxa have been associated with sPTB in women of specific ancestry (13, 14), while other women have not found significant associations (10, 15). Thus, the association between vaginal microbiota and sPTB remains unclear. It is imperative to investigate the mechanisms through which vaginal microbes stimulate the process of preterm birth.

Additionally, the concept of ascending bacterial infection and inflammation traveling from the vagina through the cervix into the uterine cavity is widely accepted and supported by both animal and human studies (3). To date, there have been a few studies have shown that bacteria ascending from the vaginal and uterine cavities can cause infection or inflammation, which are associated with sPTB (16, 17). A number of studies have revealed the association between dysbiosis of vaginal microbiota and local inflammation at the cervicovaginal interface, and these association can modulate the risk of sPTB (4, 18, 19). Recent literature also indicates that immune activation and dysregulation of immune responses occur in microbial-driven sPTB (3). However, the relationship between different subtypes of immune status and the vaginal microbiome, and their influence on the occurrence of sPTB remains unknown.

In this study, our objective was to identify diverse immune phenotypes and their characteristics in pregnant women to further clarify the immunological profiles associated with sPTB. We tested associations of cervicovaginal microbial communities and local immunological features with sPTB to discover valuable biomarkers for predicting pregnancy outcomes and identifying high-risk populations. Therefore, we utilized bidirectional intermediary analysis based on longitudinal data on vaginal microbiota and cervical-vaginal fluid (CVF) immunophenotyping, collected from 133 high-risk women with sPTB (3). We demonstrated the impact of the maternal host immune response to vaginal microbiota, and identified the mechanisms by which microorganisms influence sPTB through immunity.

## 2 Materials and methods

### 2.1 Study design and population

The data for this study was from a longitudinal pregnancy cohort in a study published by Chan et al. (3). Metadata was obtained from tables regarding sample information in the **Supplementary Material** of the article. The summarized metadata indicated whether or not the pregnant woman were premature (<37 weeks of gestation) as well as other information about the sampled subjects, mainly including age, race, time of sampling, community state type (CST), delivery time, cervical cerclage, and hormone usage. A total of 133 women, recruited from preterm birth prevention clinics in five UK Hospitals, provided a total of 385 vaginal swab samples, 126 at timepoint A (12^+0^–16^+6^ weeks), 133 at timepoint B (20^+0^–24^+6^ weeks) and 126 at timepoint C (30^+0^–34^+6^ weeks). The inclusion and exclusion criteria outlined as follows: Inclusion criteria were history of previous sPTB, previous mid-trimester loss, recurrent miscarriage, incidental finding of cervical shortening and/or cervical excisional treatment. Exclusion criteria were women under the age of 18, HIV or hepatitis C positive status, and vaginal intercourse or bleeding within 72 h of sample collection. At species level, Five CSTs were identified, four of which were dominated by a single species of *Lactobacillus*, and one which was not; CST I- *L.crispatus*, CST II - *L.gasseri*, CST III - *L.iners*, CST V - *L.jensenii*, CST IV-A reflective of high relative abundance of *BVAB1* and moderate relative abundance of *G.vaginalis*, CST IV-B reflective of a high relative abundance of *G.vaginalis* and low relative abundance of *BVAB1*, and CST IV-C reflective of low relative abundances of *G.vaginalis* and *BVAB1* (3).

The publicly available data used in this study are stored in ENA (European Nucleotide Archive), and the Accession List of the dataset was obtained according to Study Accession (PRJEB41427). The fastq sequencing files were downloaded for subsequent analysis using the “wget” command. According to the ENA Run Accession, the clinical information, 16S sequencing data in different trimesters, and immune factor expression data of each study participant were matched. Finally, the missing data were deleted.

### 2.2 Unsupervised hierarchical clustering

To identify and characterize the vaginal immune subtypes in pregnant women, we employed expression data of 22 immune factors from a large pregnancy cohort (3) for analysis. Patient’s clinical and serum data were loaded into R (v.4.0.4). Standardized scores (z-scores) for cohort were calculated based on the log10 transformed cytokine and antibody levels measured in serum. Unsupervised hierarchical clustering was performed using Ward’s Hierarchical Agglomerative Clustering Method (ward.d2) on the Euclidean distances of the z-scores. The optimal number of clusters for cohorts was assigned with the NbClust (v1.0.12) package in R. To compare the characteristics of the clusters, boxplots and Kruskal-Wallis tests were utilized. Subsequently, heatmaps were plotted using the R package pheatmap (v1.0.12).

### 2.3 16S rRNA gene sequences processing and statistical analyses

We downloaded publicly available raw 16S sequencing files of vaginal swab samples from the reported literature (3) and conducted species alignment. The raw sequencing data underwent processing and analysis utilizing the Quantitative Insights into Microbial Ecology 2 (QIIME2, version2020.2). The q2-dada2 plugin in QIIME2 was used for quality control, detection and removal of chimeras, as well as the generation of amplicon sequence variants (ASVs) along with their representative sequences. We used the SILVA database (version 138) classifier to annotate ASVs with a 99% similarity threshold. Samples with fewer than 10 ASV features or 10,000 reads, and representative sequences with frequencies below 10, were excluded from further analysis. We calculated alpha diversity using the “get_alphaindex” function from the MicrobiotaProcess R package (version 1.2.0). The Bray-Curtis distance was computed with the “vegdist” function in the vegan R package (version 2.5-7) after normalizing data through Hellinger transformation. We used the Wilcoxon rank sum test to compare alpha diversity between groups and applied permutational multivariate analysis of variance (PERMANOVA) for beta diversity comparison. Additionally, we performed Linear Discriminant Analysis Effect Size (LEfSe) using the “diff_analysis” function in the MicrobiotaProcess R package to identify differential microbiota between groups (LDA > 2). Spearman correlation analysis was conducted to examine correlations between key microbes and immune factors.

In this study, we performed the functional abundance prediction of bacterial communities based on KEGG database (http://www.genome.jp/kegg/) using PICRUSt2. While PICRUSt2 predicted the bacterial communities based on the information of the measured bacterial 16S rRNA genes and the OTU information of the proximate species after comparing with the Greengene database, and mapped the bacterial communities with the function to establish the mapping. Based on the results of PICRUSt2, STAMP software was applied to identify functional pathways with significant differences in different immune subtypes of vaginal microbiome by t-test.

### 2.4 Bi-directional mediation analysis

We employed the levels of immune factors and microbial features (abundance of differentially microbes between different immune types or pregnancy outcome groups) as candidate X or M (Mediation package) to determine the potential causal role of microbiota in preterm birth among pregnant women. Bi-directional mediation analysis: 1) Explore the relationship between X on M and X and M on Y respectively. 2) Perform mediation analysis to evaluate whether M is possible mechanisms underlying X on Y (**Supplementary Figure 1**). The effects of age and sex were corrected in each model. Finally, mediation interaction networks were constructed using Cytoscape 3.8.0.

### 2.5 *In vitro* cell co-culture experiment

Vaginal human epithelial cell lines (VK2/E6E7, ATCC CRL-2616) were cultured in Dulbecco’s Modified Eagle Medium (DMEM) high glucose (HG) supplemented with 10% Fetal Bovine Serum, 1% Penicillin-Streptomycin-Gentamicin Solution at 37 °C in a 5% CO2 humidified incubator. Human monocytic leukemia THP-1 cells were maintained in RPMI-1640, supplemented with 10% fetal bovine serum, 100 U/mL penicillin, and 100 µg/mL streptomycin.

#### 2.5.1 Bacterial cultures and preparation of bacteria-free supernatants

*G.vaginalis* (ATCC 14018) was obtained from Guangdong Microbial Culture Collection Center (GDMCC, China), while *L.crispatus* was isolated and identified from vaginal swabs taken from healthy premenopausal women without symptoms of vaginal or urinary tract infections during normal gynecological examinations. Both bacteria were inoculated onto agar plates and allowed to grow overnight. Single colonies were picked into broth medium and grown overnight again. *G.vaginalis* and *L.crispatus* were cultured in New York City (NYC) III broth in a 5% CO_2_ humidified incubator, and De Man, Rogosa, and Sharpe (MRS) broth in a bacterial incubator at 37 °C, respectively. The bacterial density of the working culture was estimated using a turbidimetric approach on the day of the experiment, and the appropriate volume was then centrifuged at 4000r, 4°C, for 5 min. The bacterial pellets were resuspended in RPMI-1640 basic cell culture media without antibiotics, and then gradient diluted to a concentration of 10^6^/mL.

To obtain *L.crispatus* supernatant, the working cultures were centrifuged at 4000r for 5 min, and the supernatant was filtered through a 0.22-μm filter (Fisher Scientific) to remove any remaining live bacteria. After overnight cultivation to confirm sterility, supernatants were diluted to 10% v/v in RPMI-1640 basic cell culture media without antibiotics.

#### 2.5.2 Cell/bacteria in transwell co-culture model

An *in vitro* non-contact co-culture model was constructed using THP-1 and VK2/E6E7 cell lines. The model involved seeding the apical side of Transwell inserts (Falcon, Corning, USA) with VK2/E6E7 cells, while THP-1 cells occupied the lower chamber. The inserts featured a pore size of 0.4 μm and were fitted to a 12-well plate. On the first day, THP-1 cells were seeded at a density of 2.5×10^4^ cells/well. THP-1 cells were induced into M_0_ macrophages by adding 10ng/mL of PMA and preincubating them in a cell culture incubator for 24 hours. Subsequently, the transwell inserts containing VK2/E6E7 cells at a density of 5×10^4^ cells per insert, were placed into Falcon 12-well plates lined with THP-1 cells. Both co-cultured cell lines were provided with their respective media and allowed to establish for 24 hours before commencing bacteria exposure studies.

We established four experimental groups, including the control group (RPMI-1640 basal medium), the *G.vaginalis*-treated group (RPMI-1640 basal medium containing *G.vaginalis*), the *L.crispatus*-treated group (RPMI-1640 basal medium containing 10% *L.crispatus* supernatant), and the *G.vaginalis* + *L.crispatus* treatment group (RPMI-1640 basal medium containing *G.vaginalis* + 10% *L.crispatus* supernatant). After establishing the co-culture model, bacterial suspensions resuspended in 1640 basal medium were added to the lower chamber as per the experimental design. The co-culture plate was subsequently placed in a CO_2_ incubator and incubated for 24 hours. Following the incubation period, the supernatant from the upper chamber was collected, and the expression levels of IL-8 and prostaglandin E2 were measured using enzyme linked immunosorbent assay (ELISA) kits.

## 3 Results

### 3.1 Identification of different immune subtypes by unsupervised hierarchical clustering

**Figure 1:**
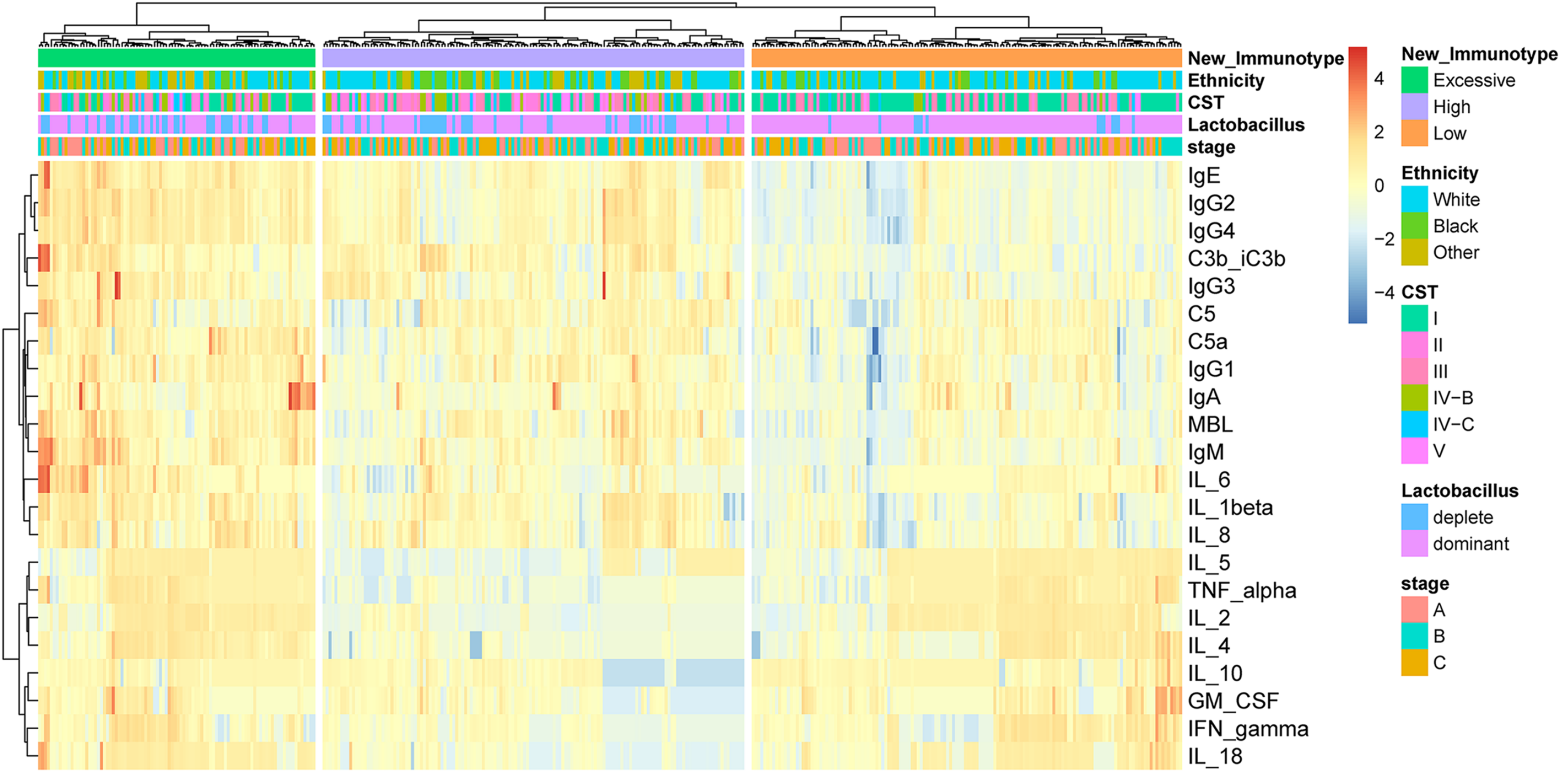
By unsupervised hierarchical clustering to immune factors identified three distinct immunotypes in pregnant woman. The three immunotypes were depicted in the heatmap of the pregnancy cohort (n = 385).

By applying unsupervised hierarchical clustering to cytokines, complement and antibodies in vaginal fluid, three distinct immune types were identified (**Figure 1**). We found the expression of each immune factor was significantly different between the three immune types (*p* < 0.05) (**Supplementary Figure 2**). Based on the expression characteristics of immune factors in the three immune types, they were respectively named as follows: High Antibody Type (HAT), Excessive Inflammatory Type (EIT), and Low Antibody Type (LAT). Immunotype HAT was characterized by low levels of anti-inflammatory cytokines and high levels of pro-inflammatory cytokines, complement, and antibodies. EIT manifested as heightened expression levels of nearly all cytokines, complement factors, and antibodies. In contrast to the previous two types, LAT exhibited markedly low levels of pro-inflammatory cytokines, complement, and antibodies.

### 3.2 Immunotype of pregnant women in mid-pregnancy is related to the occurrence of preterm birth outcomes

The chi-square test results for mid-pregnancy indicated that there were significant differences in the incidence of preterm birth among different immunotypes (**Supplementary Figure 3A**). The incidence of preterm birth was lowest in the LAT and highest in the EIT (**Table 1**). These findings suggested that EIT was associated with a higher risk of preterm birth in pregnant women, and the immune status during mid-pregnancy might serve as an indicator for preterm birth outcomes.

**Table 1:** The incidence of preterm birth among different immune phenotypes during different stages of pregnancy.

	Immunotype			*P*
	LAT	HAT	EIT	
Early pregnancy	31.60%	40%	47.80%	0.6964
Mid-pregnancy	18.20%	38.90%	87.50%	0.0091^**^
Late pregnancy	12.80%	41.70%	36.40%	0.0889

^**^ *p* < 0.01.

### 3.3 Differences in microbiota composition among pregnant women with different immunotype, and the impact of crucial microbial abundance on maternal immune status

**Figure 2:**
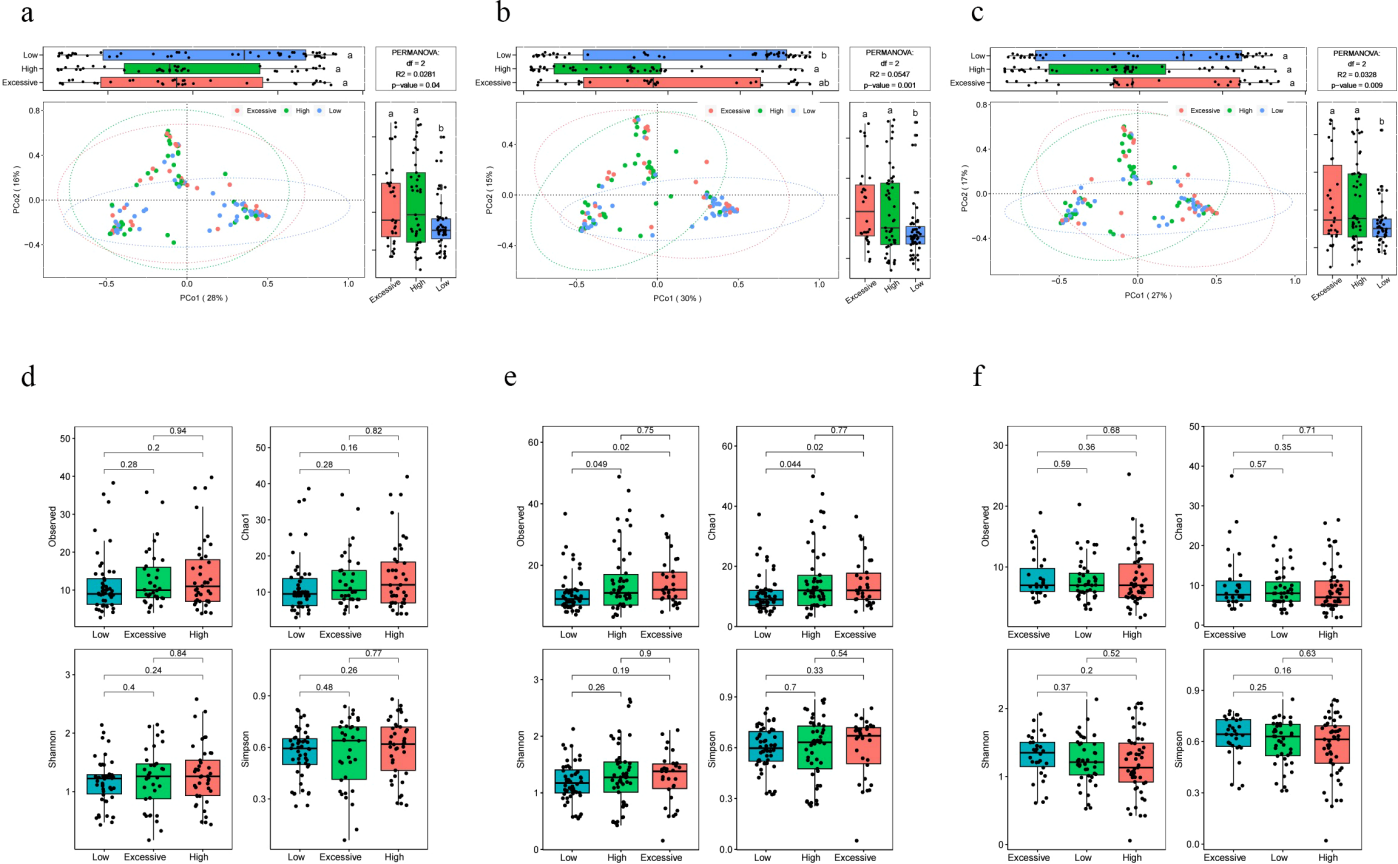
Differences in microbiota composition among pregnant women with different immunotype. **(A-C)** PCoA and boxplot are shown along the first two principal coordinates of Bray-Curtis distances for pregnant women of three immunotypes during different periods of gestation. The results of the analysis of variance are indicated by letter marks in the box plots. **(A)** early pregnancy samples, **(B)** mid-pregnancy samples, **(C)** late pregnancy samples. Ellipses represent the 95% confidence interval around the group centroid. The *P* value was calculated by PERMANOVA. **(D-F)** Four alpha diversity indicators of vaginal microbiot in pregnant women of three immunotypes during different periods of gestation. **(D)** early pregnancy samples, **(E)** mid-pregnancy samples, **(F)** late pregnancy samples. Kruskal-Wallis test was used to compare the differences between groups. The lowercase letters provided indicate significance levels of differences. Specifically, groups sharing the same letter are not significantly different, while those with different letters are significantly different. This notation helps to quickly identify which groups are statistically distinct from each other.

**Figure 3:**
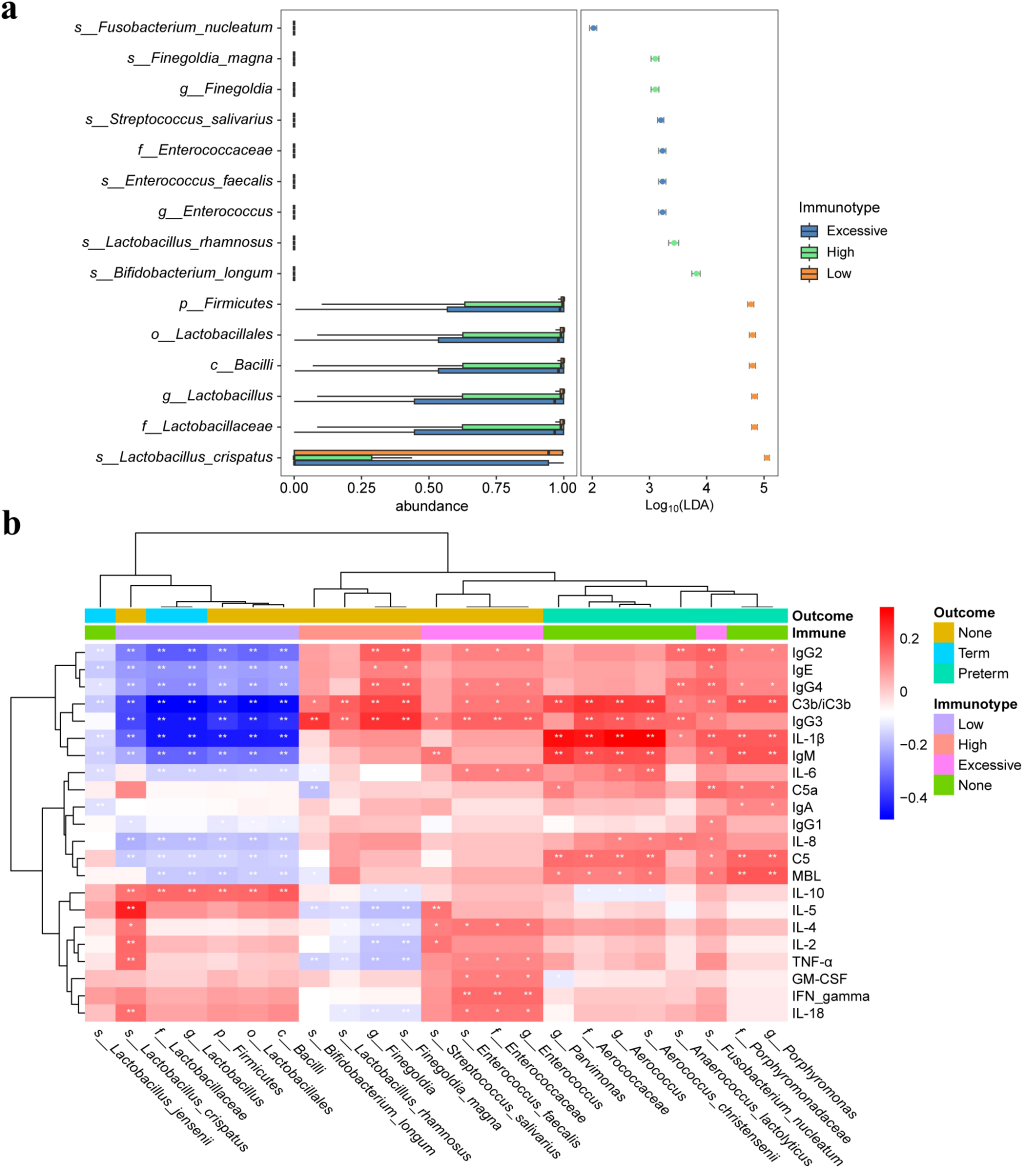
Differential microbiota and correlation of vaginal microbiota with immune factors. **(A)** Significantly different abundant taxa with LDA score (log10) > 2.0 and *P *< 0.05, between three immunotype groups. **(B)** Heatmap of Spearman correlation analysis between the differential vaginal microbiota and immune factors. Red and blue indicate positive and negative correlations, respectively. ^*^ *p* < 0.05, ^**^ *p* < 0.01.

The beta diversity analysis based on the Bray-Curtis distance revealed significant differences in vaginal microbiota composition among pregnant women with different immunotype (*P*<0.05) (**Figures 2A–C**). Specifically, there are notable differences between the LAT group and the HAT group at all three time points. Differences between the LAT group and the EIT group were observed only during the mid-pregnancy period. In early pregnancy, no significant differences were found between the EIT group and the HAT group (*P*=0.92), although trends indicating differences were noted in mid-pregnancy and late pregnancy (0.05 < *P* < 0.1) (**Supplementary Figure 5**). Regarding αdiversity, only the observed and Chao1 indices in mid-pregnancy samples show significant differences between LAT and both HAT and EIT groups, while EIT group and the HAT group are not significantly different from each other (**Figures 2D–F**). Among them, the species richness and diversity of EIT were highest and the LAT were lowest. LEfSe analysis identified 15 biomarkers that exhibited significant differences among the three immunotypes (*P*<0.05) (**Figure 3A**). The *f_Lactobacillaceae*, *g_Lactobacillus*, and *s_Lactobacillus crispatus* were significantly enriched in the LAT, while *s_Enterococcus faecalis*, *g_Enterococcus*, *s_Streptococcus salivarius*, and *s_Fusobacterium nucleatum* were significantly more abundant in the EIT. Interestingly, by comparing the results of differential bacteria analysis with those of pregnancy outcome groups, *g_Lactobacillus* and *f_Lactobacillaceae* were found to be significantly more abundant in both the LAT and the term birth group, whereas *s_Fusobacterium nucleatum* was significantly enriched in the EIT and the preterm group. In addition, correlation heatmap demonstrated a significant correlation between differential bacteria and immune factors (**Figure 3B**).

Furthermore, the analysis revealed significant differences in the proportion of different immunotypes among pregnant women with different CST types across all three stages of pregnancy. (**Supplementary Table 1**). Previous studies have shown that five CSTs were identified in the vaginal microbiome (20). CST I, dominated by *L.crispatus*, was more prone to exhibit a LAT immunotype, which associated with a lower incidence of preterm birth. In contrast, CST IV, which was diverse and low *Lactobacilli*, was more likely to exhibit a EIT immunotype with the highest incidence of preterm birth (**Supplementary Figure 3B**).

### 3.4 Differential functional pathways in the vaginal microbiota of pregnant women with different immunotypes

**Figure 4:**
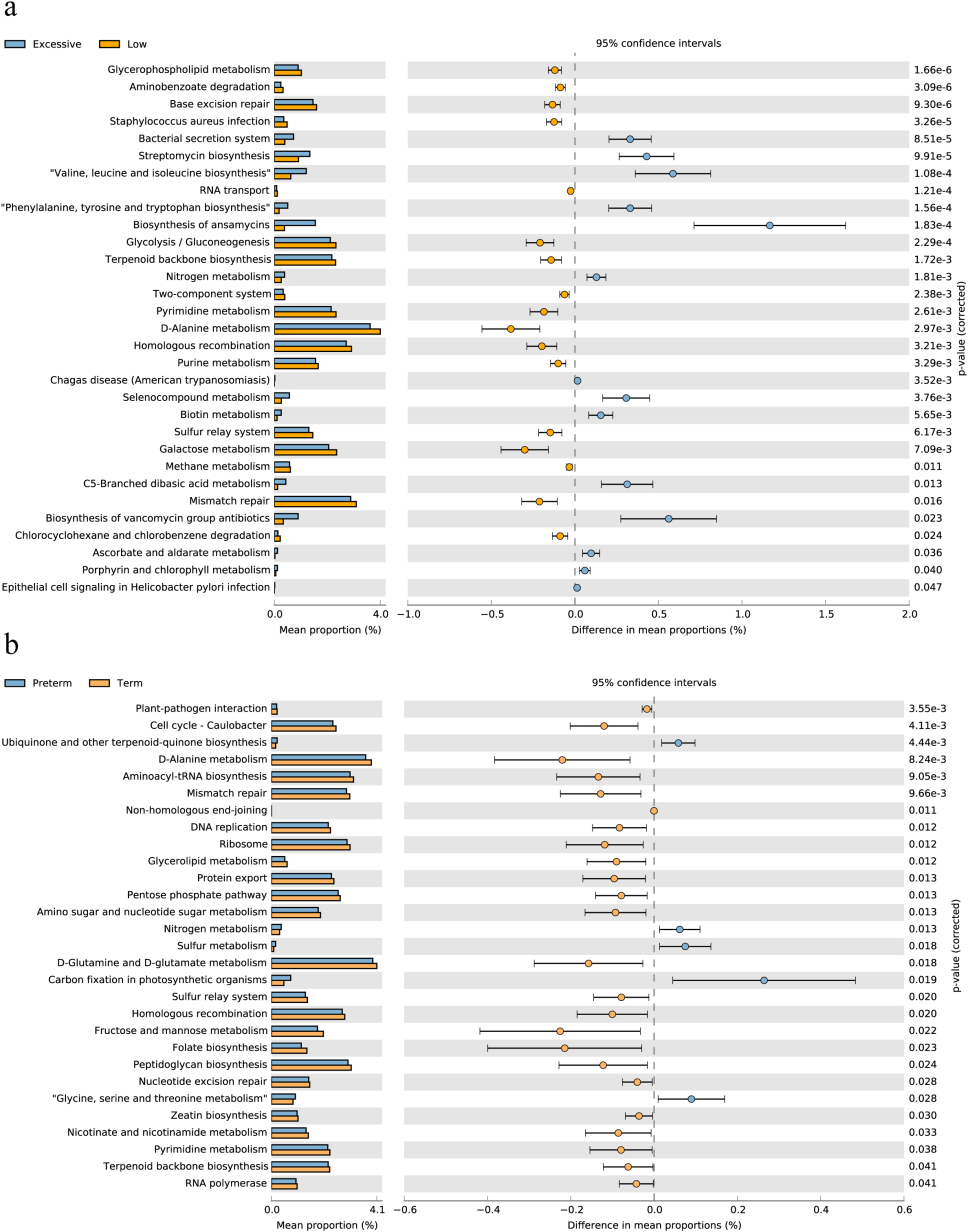
Differential functional pathways in the vaginal microbiota. **(A)** The barplot with 95% confidence intervals denote the significantly different microbial pathways between Excessive inflammatory response and Low antibody type groups. Blue, EIT; yellow, LAT. **(B)** The barplot with 95% confidence intervals denote the significantly different microbial pathways between preterm and term groups. Blue, preterm; yellow, term.

After exploring the differences in vaginal microbiome composition among women with different immunotypes, we made functional predictions. Subsequently, a differential analysis was conducted between the EIT group, which had the highest risk of preterm birth, and LAT group, which had the lowest risk. A total of 31 differential functional pathways were identified, of which 14 differential functional pathways were significantly enriched in EIT pregnant women, and 17 differential functional pathways were significantly enriched in LAT pregnant women (**Figure 4A**). Meanwhile, by comparing differential functional pathway analysis results with pregnancy outcome groups (**Figure 4B**), we identified six metabolic pathways, including *D-Alanine metabolism*, *Homologous recombination* and *Mismatch repair*, were significantly up-regulated both in the LAT and the term birth group, while *nitrogen metabolism* was significantly enriched both in the EIT and the preterm group (**Supplementary Table 2**).

### 3.5 Bi-directional mediation between the vaginal microbiota and immune factor in pregnant women affects preterm birth

**Figure 5:**
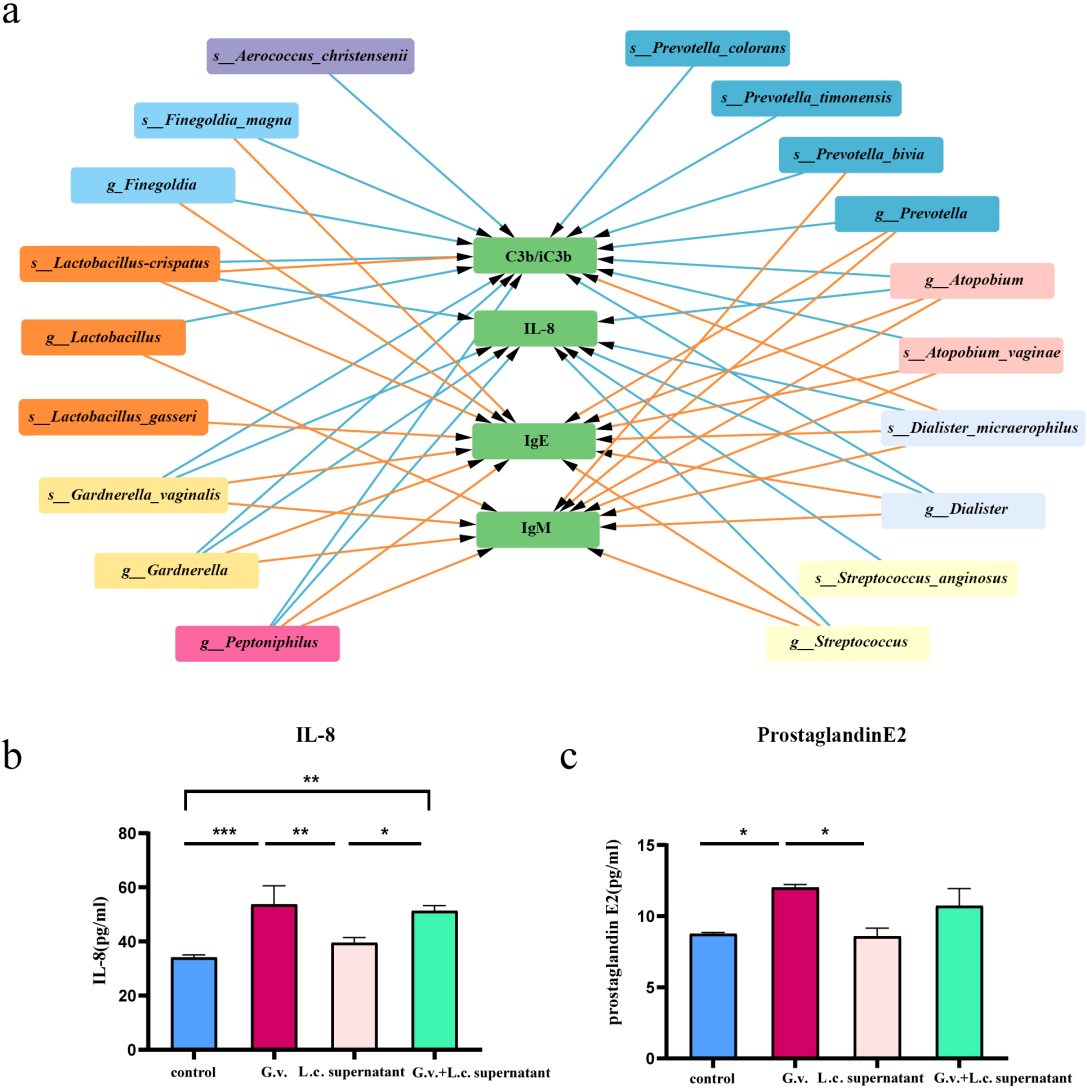
The microbe-immunity-pregnancy mediation interaction networks and experimental verification. **(A)** The microbe-immunity-pregnancy potential mediation interaction networks. Different color blocks indicate different genera of microorganisms, the green blocks indicate immunity factors, and the arrow connecting the lines indicates that the starting microorganisms may act as mediators of preterm birth outcomes through the endpoint immunity factors. The colors of the connecting lines indicate different periods, with purple representing early pregnancy, blue representing mid-pregnancy, and orange representing late pregnancy. **(B, C)** Transwell co-culture *in vitro* experiment. Histograms of IL-8 **(B)** and prostaglandin E2 **(C)** expression in *in vitro* cell co-culture experiments. Kruskal-Wallis test was used to compare the differences between groups. ^*^ *p* < 0.05, ^**^ *p* < 0.01, ^***^ *p* < 0.001.

The findings above suggested a two-by-two correlation involving immunity, microbes, and preterm birth. We further investigated whether immune factors mediate these relationships. Intriguingly, bidirectional mediation analysis revealed multiple pathways linking microbe-immunity interactions to the outcome of preterm birth. The notable microbe and immune factor mediation interaction networks were shown in **Figure 5A**. For instance, the results indicated that *L.crispatus* might avoid the occurrence of preterm birth outcomes by affecting the levels of C3b/iC3b and IL-8, whereas *G.vaginalis* might contribute to preterm delivery in pregnant women by affecting the production of IL-8. Meanwhile, we found a possible bidirectional mediating role between *L. crispatus* and the expression of immune factor C3b/iC3b, affecting the occurrence of preterm birth outcomes (**Table 2**). In summary, key vaginal bacteria are more likely to influence preterm birth outcomes by modulating vaginal immune levels.

**Table 2:** Results of Bidirectional Mediation Analysis Related to Preterm Birth Outcomes.

X	M	Intermediate effect coefficient	Gestation period
*g:Atopobium*	IL-8	0.011	mid-pregnancy
*g:Atopobium*	IgE	0.011	late pregnancy
*g:Atopobium*	IgM	0.015	late pregnancy
*g:Atopobium*	C3b/iC3b	0.021	mid-pregnancy
*g:Dialister*	IL-8	0.012	mid-pregnancy
*g:Dialister*	IgE	0.017	late pregnancy
*g:Dialister*	C3b/iC3b	0.017	mid-pregnancy
*g:Dialister*	IgM	0.021	late pregnancy
*g:Finegoldia*	IgE	0.012	late pregnancy
*g:Finegoldia*	C3b/iC3b	0.014	mid-pregnancy
*g:Gardnerella*	IgE	0.005	late pregnancy
*g:Gardnerella*	IgM	0.007	late pregnancy
*g:Gardnerella*	IL-8	0.009	mid-pregnancy
*g:Gardnerella*	C3b/iC3b	0.013	mid-pregnancy
*g:Lactobacillus*	IgM	-0.026	late pregnancy
*g:Lactobacillus*	C3b/iC3b	-0.014	mid-pregnancy
*g:Peptoniphilus*	IL-8	0.015	mid-pregnancy
*g:Peptoniphilus*	IgM	0.017	late pregnancy
*g:Peptoniphilus*	IgE	0.017	late pregnancy
*g:Peptoniphilus*	C3b/iC3b	0.026	mid-pregnancy
*g:Prevotella*	IgE	0.007	late pregnancy
*g:Prevotella*	IgM	0.011	late pregnancy
*g:Prevotella*	C3b/iC3b	0.015	mid-pregnancy
*g:Streptococcus*	IgE	0.01	late pregnancy
*g:Streptococcus*	IL-8	0.016	mid-pregnancy
*g:Streptococcus*	IgM	0.017	late pregnancy
*s:Aerococcus_christensenii*	C3b/iC3b	0.031	mid-pregnancy
*s:Atopobium_vaginae*	IgE	0.011	late pregnancy
*s:Atopobium_vaginae*	IgM	0.013	late pregnancy
*s:Atopobium_vaginae*	C3b/iC3b	0.021	mid-pregnancy
*s:Dialister_micraerophilus*	IL-8	0.016	mid-pregnancy
*s:Dialister_micraerophilus*	IgE	0.023	late pregnancy
*s:Dialister_micraerophilus*	IgM	0.027	late pregnancy
*s:Dialister_micraerophilus*	C3b/iC3b	0.043	late pregnancy
*s:Finegoldia_magna*	IgE	0.012	late pregnancy
*s:Finegoldia_magna*	C3b/iC3b	0.014	mid-pregnancy
*s:Gardnerella_vaginalis*	IgE	0.006	late pregnancy
*s:Gardnerella_vaginalis*	IgM	0.007	late pregnancy
*s:Gardnerella_vaginalis*	IL-8	0.008	mid-pregnancy
*s:Gardnerella_vaginalis*	C3b/iC3b	0.012	mid-pregnancy
*s:Lactobacillus_crispatus*	C3b/iC3b	-0.014	mid-pregnancy
*s:Lactobacillus_crispatus*	IgE	-0.008	late pregnancy
*s:Lactobacillus_crispatus*	C3b/iC3b	-0.008	late pregnancy
*s:Lactobacillus_crispatus*	IL-8	-0.007	mid-pregnancy
*s:Lactobacillus_gasseri*	IgE	0.009	late pregnancy
*s:Prevotella_bivia*	C3b/iC3b	0.016	mid-pregnancy
*s:Prevotella_bivia*	IgM	0.017	late pregnancy
*s:Prevotella_colorans*	C3b/iC3b	0.023	mid-pregnancy
*s:Prevotella_timonensis*	C3b/iC3b	0.018	mid-pregnancy
*s:Streptococcus_anginosus*	IL-8	0.016	mid-pregnancy
C3b/iC3b	*s:Lactobacillus_crispatus*	-0.02	mid-pregnancy
IL-5	*s:Lactobacillus_crispatus*	0.026	early pregnancy
C5	*s:Lactobacillus_jensenii*	-0.04	late pregnancy
C3b/iC3b	*s:Lactobacillus_jensenii*	0.014	early pregnancy
IL-10	*s:Prevotella_colorans*	0.014	late pregnancy
IgG3	*s:Streptococcus_anginosus*	-0.107	early pregnancy
IgG4	*s:Streptococcus_anginosus*	-0.066	early pregnancy

### 3.6 *In vitro* co-culture experiments provided supportive evidence that microorganisms could affect the occurrence of preterm birth through modulation of immune responses

To validate the results of bidirectional mediation analysis, we conducted transwell co-culture experiments *in vitro*. We selected THP-1 cell line as the mediator cells responsible for secreting immune factors to detect the differential expression of mediator factors. VK2/E6E7 cells were chosen as the indicator cells for preterm birth outcomes, and the differential expression of prostaglandin E2 by VK2/E6E7 cells indirectly reflected the status of vaginal epithelial cells, indicating the occurrence of preterm birth. As *G.vaginalis* is a commonly found pathogenic bacterium in the female reproductive tract and is associated with an unhealthy vaginal microbiota composition, along with numerous reports indicating its significant enrichment in women with preterm birth (21, 22), we intended to validate the *G.vaginalis* - IL-8 - preterm delivery pathway suggested by the results of the mediation analysis. Furthermore, we also validated the inhibitory effect of *L.crispatus* on preterm birth outcomes.

Our experimental results revealed significant differences between the *G.vaginalis*-treated group and the *G.vaginalis* + *L.crispatus* treatment group compared to the control group after 24 hours of co-culture. The expression levels of IL-8 and prostaglandin E2 were significantly higher in the *G.vaginalis*-treated group than in the control group (**Figures 5B, C**), suggesting that *G.vaginalis* was able to induce the secretion of IL-8 by immune cells. Although the difference between the *G.vaginalis* + *L.crispatus* treatment group and *G.vaginalis*-treated group was not statistically significant, the expression levels of IL-8 and prostaglandin E2 were lower than those of the *G.vaginalis*-treated group, suggesting that *L.crispatus* supernatant may reduce the stimulatory effect of *G.vaginalis* on the secretion of IL-8 by immune cells. In addition, the results showed that IL-8 was positively correlated with the expression of prostaglandin E2 (r = 0.6503) (**Supplementary Figure 4**), an effector molecule associated with preterm birth, suggesting that high expression of IL-8 may lead to vaginal epithelial remodeling and accelerate the process of labor.

## 4 Discussion

In this study, we aimed to investigate the relationship between vaginal immune status and microbiome composition the risk of preterm birth. Our findings highlight several novel insights that contribute to the understanding of PTB pathogenesis and potentially inform clinical strategies for its prevention. Recent research suggests that changes in the vaginal microbiome help predict the risk of spontaneous preterm birth (4, 18, 23). However, the mechanisms by which genital microbiota influence the birthing process remain poorly understood. Experimental studies aiming to manipulate the composition of vaginal flora through probiotic interventions have yielded varied and inconsistent results (24–27). Therefore, we propose that there might be key factors connecting microbes to preterm birth outcomes, which play a critical role in the overall process. Our findings build upon and extend previous research in this field, which has primarily focused on individual microbial species or immune factors in isolation. By integrating both immune status and microbiome composition, we provide a more comprehensive view of the vaginal environment and its role in PTB. Our results suggest that alterations in both the immune system and microbiome contribute synergistically to PTB risk, a perspective that is novel and adds depth to the current scientific literature.

As shown in previous study (28), we distinctly categorized three immune types associated with changes in immune levels and pregnancy outcomes by employing unsupervised clustering of data on 22 immune factors. Notably, there were notable differences in preterm birth rates among the three immune types during mid-pregnancy, with the EIT type showing active immune responses and a higher incidence of preterm birth. This also suggested that the vaginal local immune status of pregnant women in mid-pregnancy could serve as an indicator of preterm birth outcomes, and that elevated immune levels may contribute to preterm birth. Recent studies revealed that high vaginal bacterial load during the middle of pregnancy was associated with sPTB in high-risk pregnant women (12, 29). These findings consistent with our results, suggest that the mid-pregnancy may be a crucial period for predicting subsequent preterm labor events.

Our study has indicated different in the diversity and composition of the vaginal microbiota among all three groups during mid-pregnancy. Specifically, pregnant women categorized as EIT displayed the highest species abundance and diversity, while those classified as LAT exhibited the lowest. In asymptomatic, otherwise healthy women, the majority often dominated by species of Lactobacillus. Changes in the vaginal microbiota diversities including reduced Lactobacillus abundance and increased facultative and anaerobic organism populations could predisposes the host to several conditions like increased risk of contracting bacterial infections (30). This pro-inflammatory climate created by dysbiosis of the microbiota could induce secondary inflammatory mediators via Toll-like receptors (31). This may be the reason why the excessive group and high group both showed similar higher alpha diversities and pro-inflammatory cytokines. Prior research has also demonstrated that antigen-presenting cells in the genital tract can mount specific responses to Gram-negative bacterial products via Toll-like receptor signaling. This activation triggers the NF-κB signaling pathway, initiating the recruitment of lymphocytes by chemokine production, ultimately leading to inflammation in the genital tract (32). Thus, the above results indicate a potential correlation between vaginal flora composition and local immune status. It can be speculated that certain bacteria in the genital tract may induce robust local immune responses.

The correlation analysis revealed that communities with high diversity showed a strong correlation with pro-inflammatory cytokine concentrations in the genital tract. The results of the bidirectional mediation analysis provided stronger evidence supporting the hypothesis that microbiota influences preterm birth through immunity. Forward mediation analysis suggested that specific microorganisms, such as *Atopobium vaginae*, *Aerococcus christensenii*, and *Gardnerella vaginalis*, could influence the occurrence of preterm birth outcomes by modulating the levels of immune factors. In contrast, the reverse mediation analysis only identified a few mediating pathways, indicating that immune factors primarily play a mediating role. Some of the key microorganisms we identified have been linked to preterm birth. For instance, Odogwu et al. (29) found that the relative abundance of *Atopobium vaginae* was higher in mid-pregnancy and strongly predictive of preterm birth onset. Additionally, Doyle et al. (33) discovered that *Aerococcus christensenii* and *Gardnerella vaginalis* were more prevalent in preterm placental membranes. We believe that these key microorganisms are related to the immune factors activation, which may prematurely activate the delivery procedure and induce preterm birth (34). Moreover, we observed that the mediating immune factors that played a key role in microbial-induced preterm birth in mid-gestation were C3b/iC3b and IL-8, whereas in late gestation they were IgE and IgM. We hypothesized that this temporal specificity may be related to the differences in the speed of innate and specific immune responses. Differences in maternal host immune recognition and response to vaginal microbial communities may explain why only a subset of women with high-risk vaginal microbial features undergo preterm birth. In addition, the experimental results showed that the expression of IL-8 as well as prostaglandin E2 in the *G.vaginalis*-treated group was significantly higher than that in the control group, whereas the *G.vaginalis*+*L.crispatus* treatment group was lower than that of the *G.vaginalis*-treated group, but the difference was not statistically significant. Therefore, we concluded that *G.vaginalis* could induce the secretion of IL-8 by immune cells and promote vaginal epithelial remodeling.

The study findings indicated that the presence of live *Gardnerella vaginalis* and *Lactobacillus crispatus* supernatant may trigger a specific epithelial immune response, breaching the cervical vaginal epithelial barrier. Although a single-cell model enables detailed exploration of the fundamental mechanisms through which epithelial immune responses are modulated in the presence of specific microbial species, it is unable to fully replicate the authentic conditions of the pregnant female reproductive tract. We acknowledge that the lack of statistical significance could potentially stem from limitations in our cellular experiment design, such as bacterial concentration, co-culture duration, and cell numbers. To address these concerns and provide stronger evidence for our hypothesis, we believe a more refined experimental design is necessary for further validation.

To translate our findings into clinical practice, further validation studies in larger, more diverse populations are needed. Once validated, the identified immune markers and microbial signatures could be incorporated into routine prenatal screening protocols. This would enable clinicians to identify women at risk of PTB early on, allowing for timely interventions such as intensified monitoring, prophylactic antibiotics, or immunomodulatory therapies, where appropriate.

Furthermore, our findings underscore the importance of maintaining a healthy vaginal microbiome during pregnancy. This highlights the potential for probiotics, prebiotics, or other microbial-based therapies to be explored as preventive measures against PTB. Clinical trials evaluating the efficacy of such interventions in reducing PTB risk are warranted.

Therefore, the establishment of an association between vaginal microbial profiles and PTB risk opens up new avenues for preventive strategies and early detection methods. Meanwhile, our work contributes to the growing body of literature that emphasizes the intricate interplay between the vaginal microbiome, host immunity, and pregnancy outcomes. Our findings suggest that the vaginal microbiome and immune status could potentially serve as biomarkers for identifying women at heightened risk of PTB. However, translating this concept into clinical practice necessitates further investigation into the feasibility, accuracy, and cost-effectiveness of implementing such screening programs.

## 5 Conclusions

The study holds significant implications for the clinical management of pregnant women by identifying specific signatures combining immune and microbial factors associated with sPTB. Our research revealed an association between local vaginal immune status and the vaginal microbiome. Furthermore, microorganisms were found to influence preterm birth by modulating immune factors levels, with time-specific mediation effects observed. Based on our bidirectional mediation analysis, we selected a pathway for validation through *in vitro* cell experiments. This helped elucidate the microbiome-immunity-sPTB axis and confirming the reliability of our findings. Finally, *L.crispatus* demonstrated potential in protecting against sPTB by modulating vaginal immune status.

## Data Availability

The data presented in the study are deposited in the National Omics Data Encyclopedia (NODE; https://www.biosino.org/node/index) repository, accession number OEP004691.

## Supplementary material

Supplementary Figure 1 Steps in bi-directional mediation analysis.

Supplementary Figure 2 Expression characteristics of 22 immune factors in the three immunotypes. The boxplots and Kruskal-Wallis test were used to compare the differences between groups. ^*^ *p* < 0.05, ^**^ *p* < 0.01, ^***^ *p* < 0.001.

Supplementary Figure 3 Incidence of premature birth and the characteristics of vaginal microbiota in mid pregnancy. **(A)** The incidence of preterm birth among different immunotypes during mid-pregnancy. **(B)** The proportion of different immunotype within each CST during mid-pregnancy. ^*^ *p* < 0.05, ^**^ *p* < 0.01, ^***^ *p* < 0.001.

Supplementary Figure 4 Spearman correlation between expression of IL-8 and prostaglandin E2 in transwell co-culture *in vitro* experiment.

Supplementary Figure 5 The PCoA and boxplot display the first two principal coordinates of Bray-Curtis distances, facilitating pairwise comparisons of the three immunotypes in pregnant women across different gestational periods. **(A-C)** early pregnancy samples, **(D-F)** mid-pregnancy samples, **(G-I)** late pregnancy samples. Ellipses represent the 95% confidence interval around the group centroid. The P value was calculated by PERMANOVA.
