# Corrigendum: Modulation of local immunity by the vaginal microbiome is associated with triggering spontaneous preterm birth

**DOI:** 10.3389/fimmu.2024.1544081

**Published:** 2025-01-06

**Authors:** Yijia Liang, Changying Zhao, Yan Wen, Dashuang Sheng, Tiantian Wei, Tianqi Hu, Junhui Dai, Guoping Zhao, Sijie Yang, Qinghua Wang, Lei Zhang

**Affiliations:** ^1^ Microbiome-X, School of Public Health, Cheeloo College of Medicine, Shandong University, Jinan, China; ^2^ Reproductive Medicine Center, Shandong Provincial Maternal and Child Health Care Hospital Affiliated to Qingdao University, Jinan, China; ^3^ Qingdao West Coast New District Health Bureau, Qingdao, China; ^4^ State Key Laboratory of Microbial Technology, Shandong University, Qingdao, China; ^5^ CAS Key Laboratory of Computational Biology, Bio-Med Big Data Center, Shanghai Institute of Nutrition and Health, University of Chinese Academy of Sciences, Chinese Academy of Sciences, Shanghai, China; ^6^ State Key Laboratory of Reproductive Medicine and Offspring Health, Center for Reproductive Medicine, Institute of Women, Children and Reproductive Health, Shandong University, Jinan, China; ^7^ School of Biological Science and Technology, University of Jinan, Jinan, China

**Keywords:** preterm birth, vaginal microbiome, immunity, *Gardnerella vaginalis*, *Lactobacillus crispatus*

In the published article, there was an error in affiliation 7. Instead of “Department of Microbiology, School of Public Health, Cheeloo College of Medicine, Shandong University, Jinan, China”, it should be “School of Biological Science and Technology, University of Jinan, Jinan, China”.

The authors apologize for this error and state that this does not change the scientific conclusions of the article in any way. The original article has been updated.

