# Supplementary figures and images for: Modulation of local immunity by the vaginal microbiome is associated with triggering spontaneous preterm birth
Source: Front Immunol. 2024 Nov 18;15:1481611. doi: 10.3389/fimmu.2024.1481611 (PMC11609181; doi:10.3389/fimmu.2024.1481611)

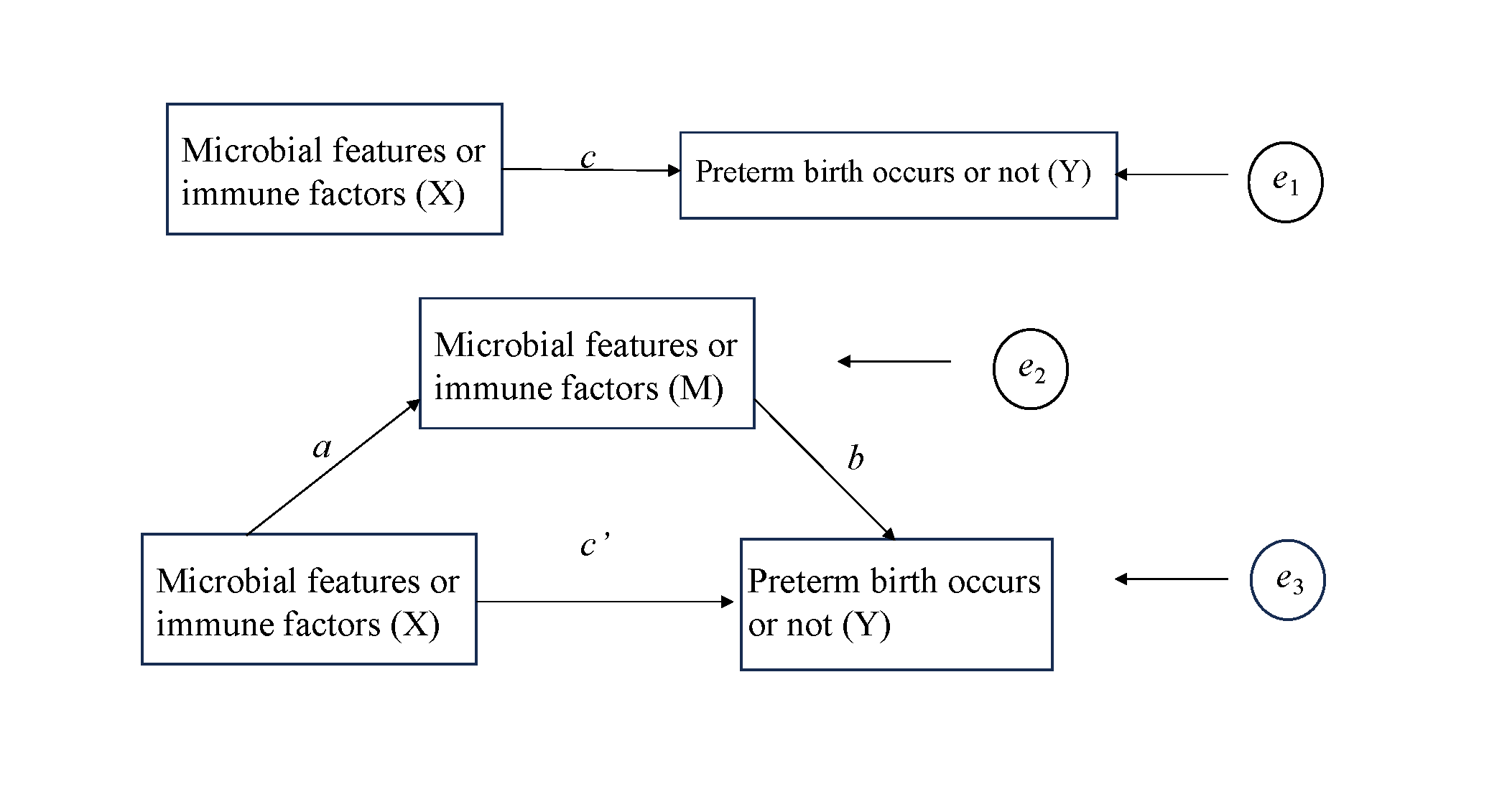

Supplement: Supplementary Figure 1 — Steps in bi-directional mediation analysis. [file Image1.tif]

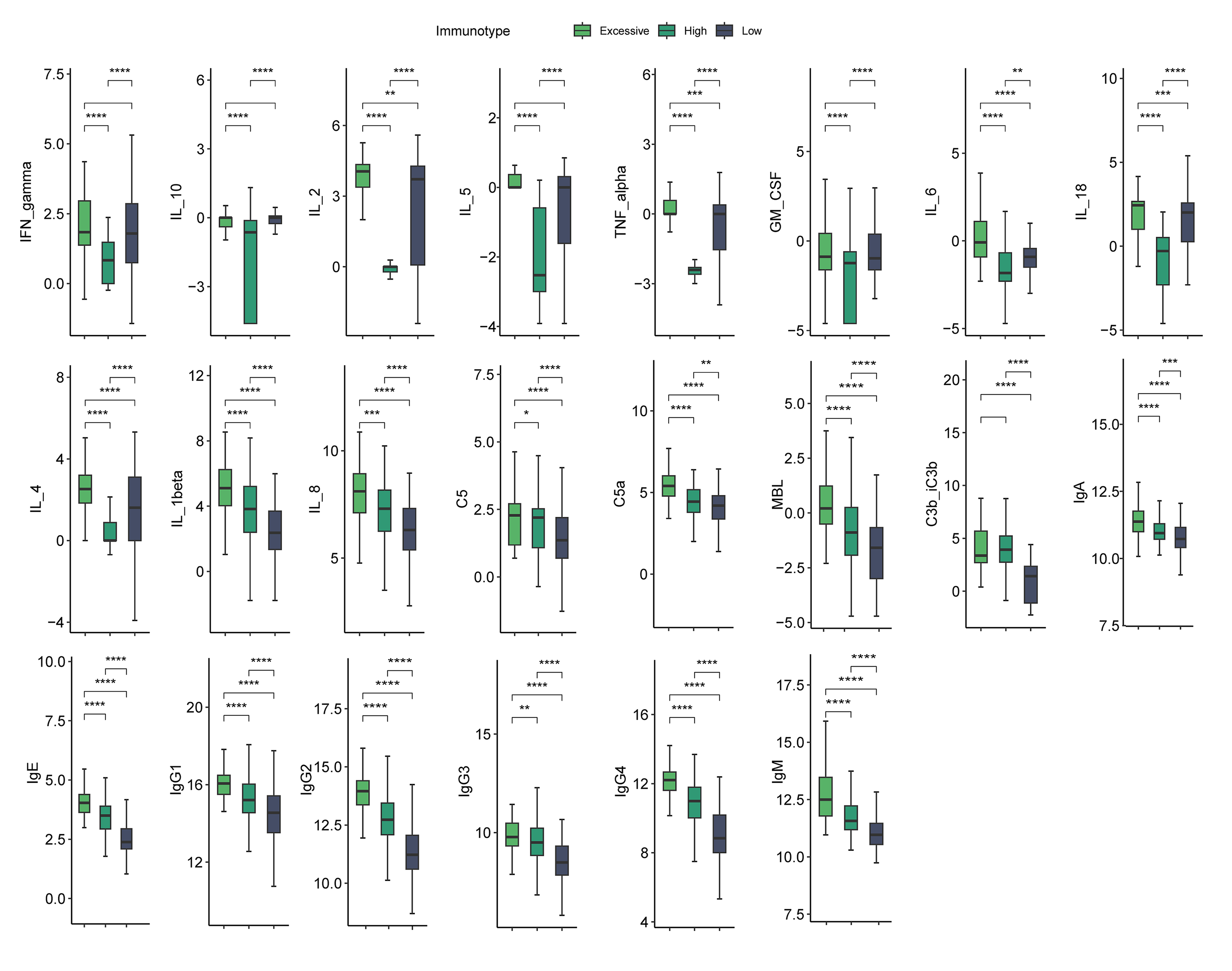

Supplement: Supplementary Figure 2 — Expression characteristics of 22 immune factors in the three immunotypes. The boxplots and Kruskal-Wallis test were used to compare the differences between groups. * p < 0.05, ** p < 0.01, *** p < 0.001. [file Image2.tif]

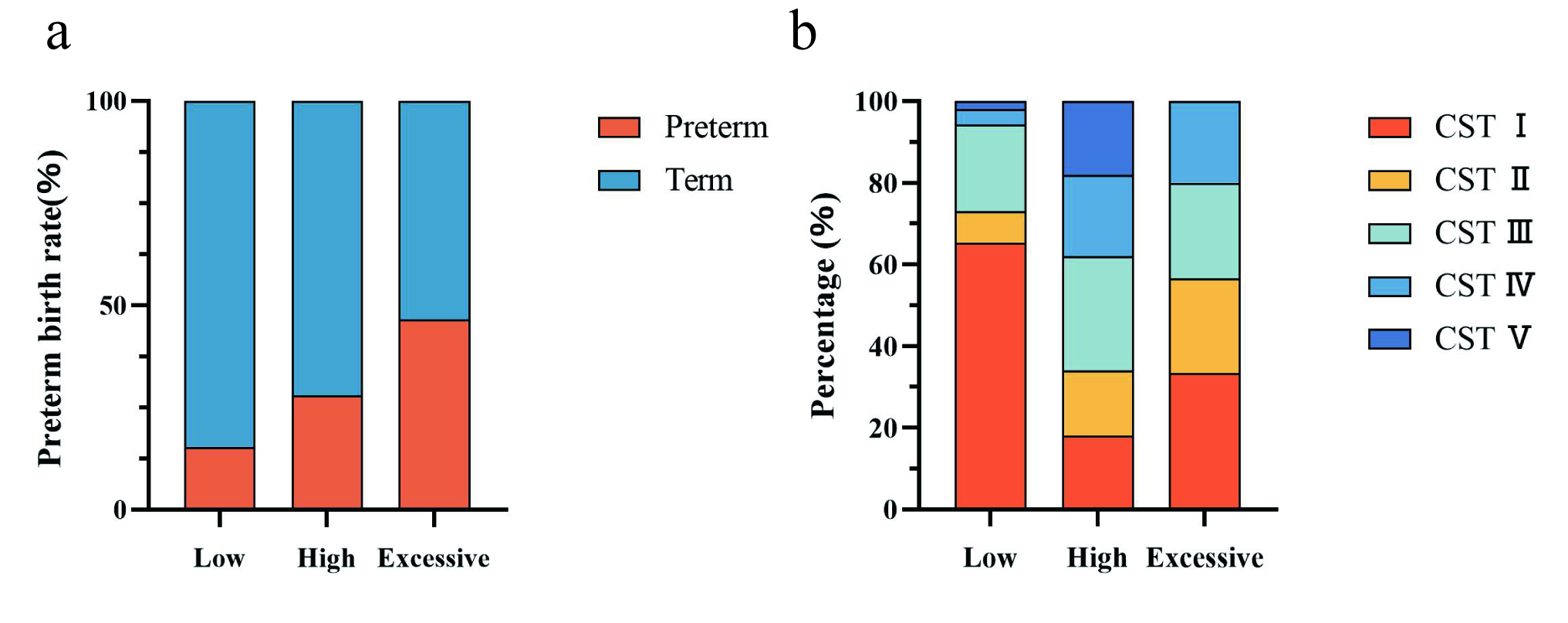

Supplement: Supplementary Figure 3 — Incidence of premature birth and the characteristics of vaginal microbiota in mid pregnancy. (A) The incidence of preterm birth among different immunotypes during mid-pregnancy. (B) The proportion of different immunotype within each CST during mid-pregnancy. * p < 0.05, ** p < 0.01, *** p < 0.001. [file Image3.tif]

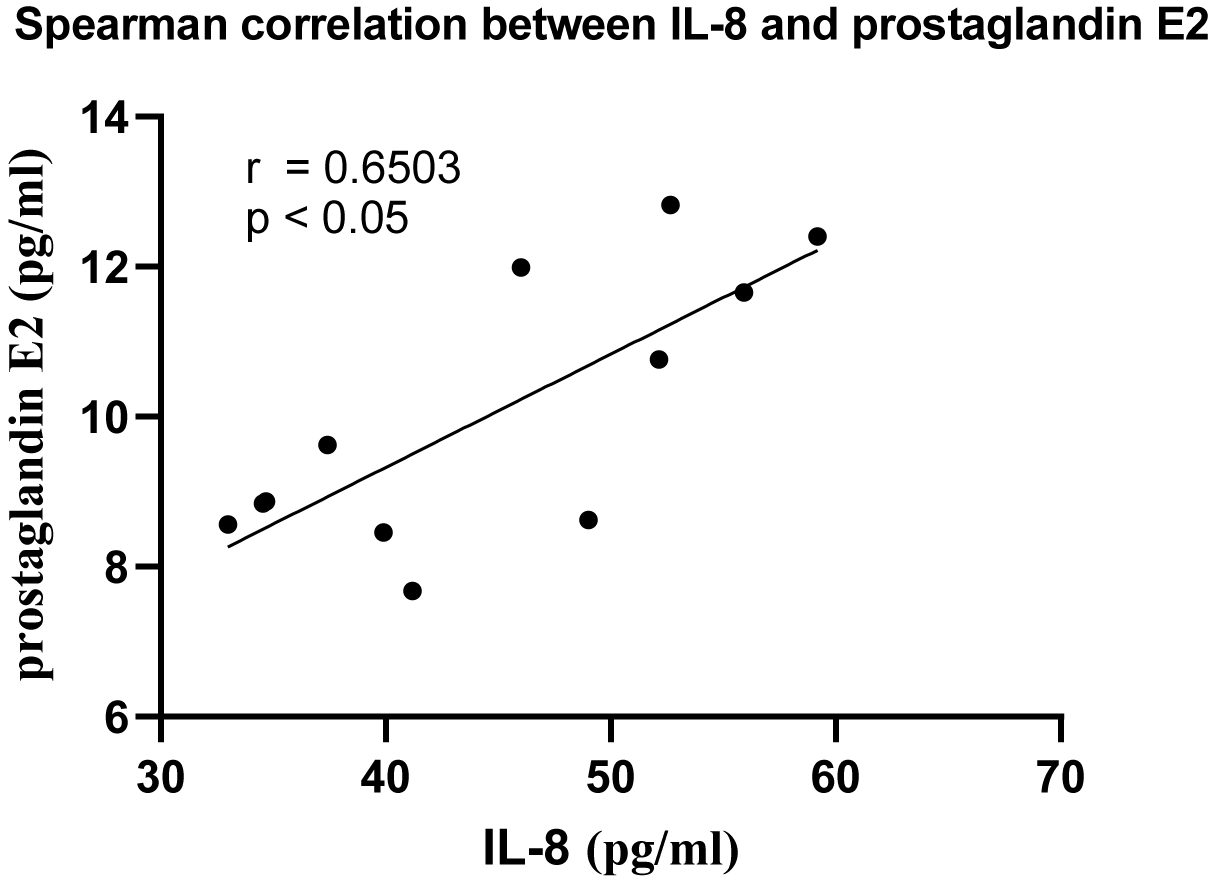

Supplement: Supplementary Figure 4 — Spearman correlation between expression of IL-8 and prostaglandin E2 in transwell co-culture in vitro experiment. [file Image4.tif]

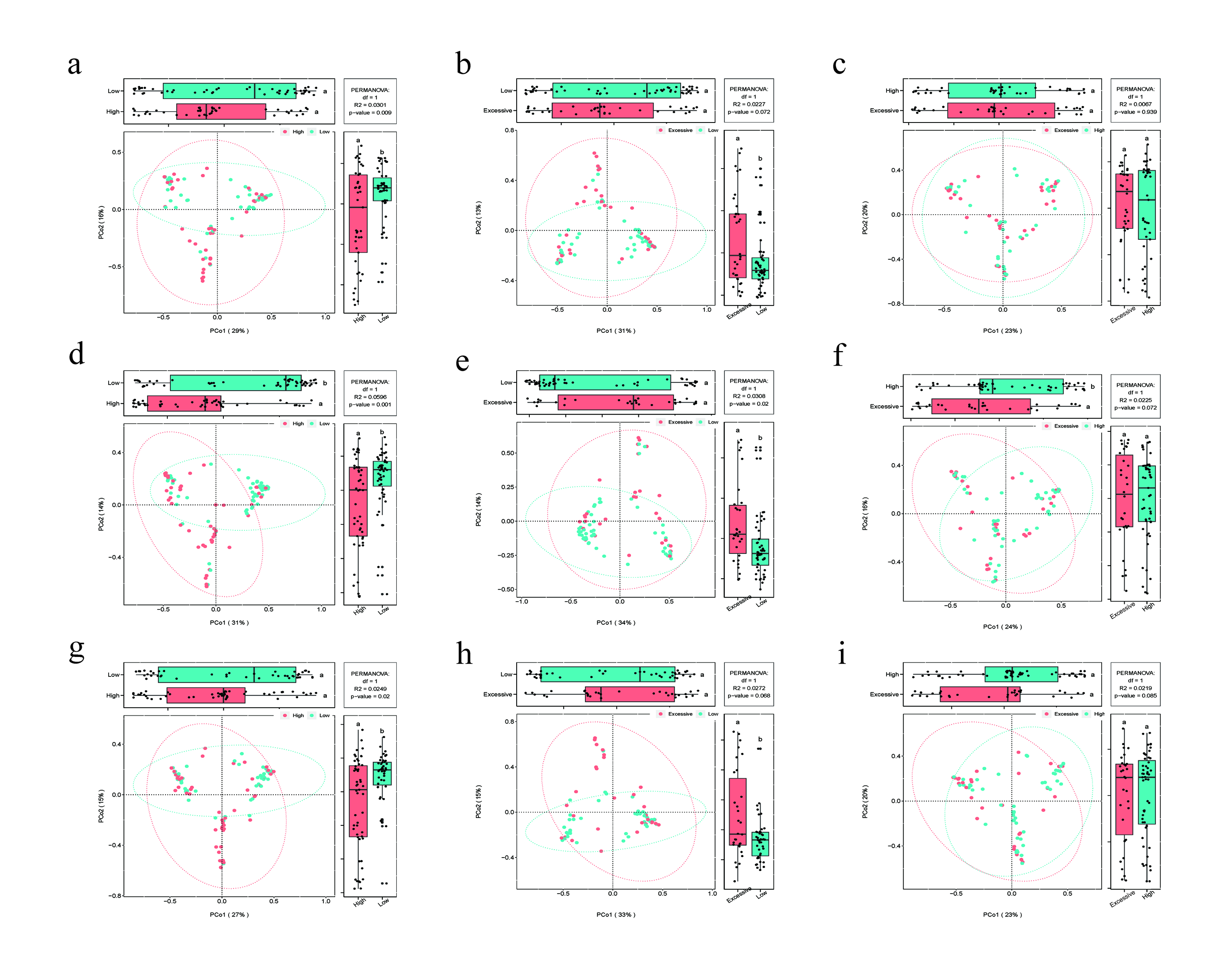

Supplement: Supplementary Figure 5 — The PCoA and boxplot display the first two principal coordinates of Bray-Curtis distances, facilitating pairwise comparisons of the three immunotypes in pregnant women across different gestational periods. (A-C) early pregnancy samples, (D-F) mid-pregnancy samples, (G-I) late pregnancy samples. Ellipses represent the 95% confidence interval around the group centroid. The P value was calculated by PERMANOVA. [file Image5.tif]
